# Identifying lncRNA–disease association based on GAT multiple-operator aggregation and inductive matrix completion

**DOI:** 10.3389/fgene.2022.1029300

**Published:** 2022-10-20

**Authors:** Yi Zhang, Yu Wang, Xin Li, Yarong Liu, Min Chen

**Affiliations:** ^1^ Guilin University of Technology, Guilin, China; ^2^ Guangxi Key Laboratory of Embedded Technology and Intelligent System, Guilin University of Technology, Guilin, China; ^3^ School of Computer Science and Technology, Hunan Institute of Technology, Hengyang, China

**Keywords:** graph attention network, inductive matrix completion, association prediction, aggregation, multiple-operator

## Abstract

Computable models as a fundamental candidate for traditional biological experiments have been applied in inferring lncRNA–disease association (LDA) for many years, without time-consuming and laborious limitations. However, sparsity inherently existing in known heterogeneous bio-data is an obstacle to computable models to improve prediction accuracy further. Therefore, a new computational model composed of multiple mechanisms for lncRNA–disease association (MM-LDA) prediction was proposed, based on the fusion of the graph attention network (GAT) and inductive matrix completion (IMC). MM-LDA has two key steps to improve prediction accuracy: first, a multiple-operator aggregation was designed in the n-heads attention mechanism of the GAT. With this step, features of lncRNA nodes and disease nodes were enhanced. Second, IMC was introduced into the enhanced node features obtained in the first step, and then the LDA network was reconstructed to solve the cold start problem when data deficiency of the entire row or column happened in a known association matrix. Our MM-LDA achieved the following progress: first, using the Adam optimizer that adaptively adjusted the model learning rate could increase the convergent speed and not fall into local optima as well. Second, more excellent predictive ability was achieved against other similar models (with an AUC value of 0.9395 and an AUPR value of 0.8057 obtained from 5-fold cross-validation). Third, a 6.45% lower time cost was consumed against the advanced model GAMCLDA. In short, our MM-LDA achieved a more comprehensive prediction performance in terms of prediction accuracy and time cost.

## Introduction

Long non-coding RNA, named for its transcription length of over 200 nucleotides, has received extensive attention from biological researchers (Sun et al., 2018). With the in-depth development of biomedicine, many literatures have confirmed that lncRNA plays an important role in the activities of living organisms through dose compensation effect, genetic expression, cell differentiation, and other ways and gradually becomes the focus of bioinformatics. Studies have shown that abnormal lncRNA expression can lead to a variety of complex diseases, especially as both oncogenes and tumor suppressors in the tumorigenesis of diverse cancers (Chen et al., 2020). The exploration of lncRNA leading to disease is helpful in understanding the mechanism of disease generation and provides reference for disease treatment and prognosis (Xia et al., 2013). Therefore, the work on predicting lncRNA–disease associations is significant for human disease diagnostics and prognostics and will improve the development of drug discovery (Chen et al., 2020).

As biological experiments are time-consuming and laborious, numerous computational models are mostly used to replace biological experiments in real life to identify disease-related associations and provide efficient and more accurate candidates for biological experiments in recent years (Chen et al., 2019; Wang et al., 2021; Huang et al., 2022a; Huang et al., 2022b; Huang et al., 2022c). Currently, computational models for predicting lncRNA–disease associations (LDAs) commonly fall into three categories.

The first category of methods is based on constructing biological similarity networks. Label propagation algorithms are used commonly in association-related prediction (Yin et al., 2020), especially as restart random walk and KATZ, whose main difference is applied in different underlying networks. Sun et al, (2014) and Chen et al, (2016) established the global restart random walk algorithm by using the lncRNA functional similarity network so as to predict potential association information. However, these models could not work on isolated diseases (diseases without known association information) or new lncRNAs (lncRNAs without known association information). Based on the gene–disease association and lncRNA–disease similarity network, Ma et al, (2019) introduced the HeteSim algorithm to construct a gene–disease heterogeneous information network, with which the network structure was strengthened by increasing the number of edges in the network. Potential associations can be propagated with more information and with better prediction effects. Chen, 2015; Chen et al, (2019) combined known LDA, lncRNA expression profile information, lncRNA functional similarity, disease semantic similarity, and Gaussian interaction spectrum kernel similarity to establish association prediction models. Although these models could work on isolated diseases or new lncRNAs, the prediction accuracy is still not high enough.

The second category of methods utilizes machine learning with a classifier to identify pathogenic lncRNAs. Chen and Yan, (2013) used lncRNA expression profile information to develop a classic and significant calculation model LRLSLDA for inferring potential lncRNA–disease pair information. This model is the first to use Laplacian regularized least squares in a semi-supervised learning framework, and it could work on new lncRNAs and isolated diseases without needing negative samples. However, its selection of optimal parameters is complicated because of its disease space and lncRNA space belonging to two classifiers. Later, Chen et al, (2015) developed an improved correlation prediction model LNCSIM to further improve the prediction accuracy. However, with its prediction results biased toward those lncRNAs with more known associations, the prediction effect is not good enough for isolated diseases and new lncRNAs with less known information. In addition, selecting attenuation factors of semantic contribution has not been well-solved. Zhao et al, (2015) predicted potentially pathogenic lncRNA by integrating known disease-related lncRNA and a variety of biological data (genomic data, regulatory, and transcriptional biological data) based on the Bayesian algorithm. Although the prediction performance of this model is good, sufficient negative samples of the Bayesian classifier are required to improve the prediction performance.

The third category of methods is based on disease-related genes, for example, mRNA, miRNA, and protein information. Models belonging to the aforementioned two categories all rely on the known LDA, whose number with experimental verification is relatively small. Therefore, researchers have to explore new ideas to infer the potential associations with using third-party data, also known as genetic information. Zhou et al, (2015) selected appropriate thresholds and coefficients to predict lncRNA–disease pairs, using the expression data of three kinds of non-coding RNAs (mRNA, miRNA, and lncRNA). Cheng et al, (2016) introduced mRNA- and miRNA-related data into the prediction of LDA. Compared with other methods, methods within this category are more reliable and stable, but the model performance is highly dependent on coactions found among the three kinds of non-coding RNAs.

**FIGURE 1:**
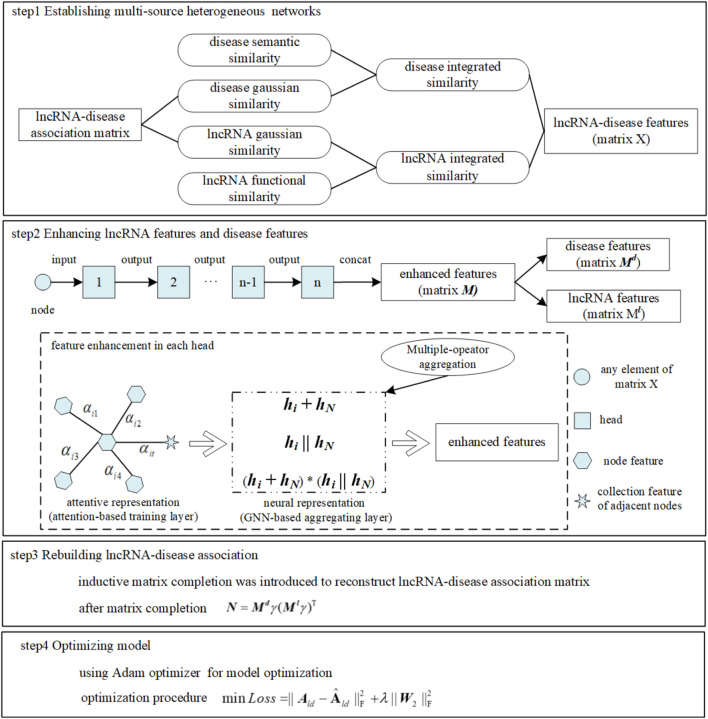
MM-LDA workflow.

Utilizing deep learning technology has gradually become a research hotspot to make up for the deficiencies in the abovementioned three categories. The graph that can abstract the relationship between entities is widely used as a data structure (Wu et al., 2020). Wu et al, (2021) proposed a computational method MLGCNET that applied the graph convolutional network (GCN) to extract the node information with which to feed into an extra tree (ET) classifier for accurately predicting the potential lncRNA–disease associations. The graph attention network (GAT), as a promising graph neural network, has been applied to a number of bioinformatics tasks. Long et al, (2021) proposed a new method GATMDA based on the GAT to identify a microbial–disease association. Bian et al, (2021) proposed a model GATCDA to predict circRNA–disease associations based on the GAT. Gu et al, (2021) predicted drug ADMET classification based on the GAT. However, this model did not discuss the time complexity consumed for achieving high accuracy. Inductive matrix completion (IMC) that could fill data sparsity existing in the bio-database inherently caused the problem of low prediction accuracy when it was applied in inferring LDA directly and separately (Natarajan and Dhillon, 2014; Huang et al., 2017; Chen et al., 2018; Lu et al., 2018; Fraidouni and Zaruba, 2019; Chen et al., 2021). Therefore, to break through the aforementioned limitations, multiple mechanisms were fused into a new computational model, such as MM-LDA, as shown in Figure 1. On one hand, a multiple-operator aggregation used in the n-heads attention mechanism of the GAT was designed, where it could enhance the features of lncRNA nodes (or disease nodes) to avoid the low prediction accuracy caused by known-data sparsity. On the other hand, with enhanced node features, the LDA network was rebuilt by IMC that could renew the missing elements in the bio-database. In the end, the Adam optimizer was used to further improve the prediction accuracy.

## Materials and methods

### Data source

Known lncRNA–disease association: After removing repeated and redundant lncRNAs (diseases) in the original dataset lncRNA disease V2.0 (Bao et al., 2019), a processed dataset composed of associations between human diseases and lncRNAs was used in our model. This dataset contains 352 LDAs verified experimentally, involving 156 lncRNAs and 190 diseases. It is an unbalanced dataset with existing inherent data sparsity because of less known associations against unknown or non-existent associations.

For formal description later, the number of lncRNAs and diseases involved in this dataset (also called association matrix) was denoted by nl and nd , respectively. In the association matrix ( $A_{ld}\in R^{nl\times nd}$ ), any known lncRNA–disease association that relates to disease $d_{i}$ and lncRNA $l_{j}$ with experimental verification works as the positive sample, with denotation of $A_{ld}\left(l_{i},d_{j}\right)=1$ . Otherwise, any unknown or non-existent lncRNA–disease association works as the negative sample, with denotation of $A_{ld}\left(l_{i},d_{j}\right)=0$ .

### Multi-source heterogeneous networks

Disease–disease semantic similarity network: Directed acyclic graph (DAG) was utilized to calculate the semantic similarity between diseases (Wang et al., 2010). The semantic contribution value of any disease $d_{t}$ to disease $d_{i}$ was denoted by $D_{d_{i}}\left(d_{t}\right).$

$$D_{d_{i}}\left(d_{t}\right)=\left\{ \begin{array}{cc} 1, & d_{t}=d_{i},\\ \max\left\{\gamma D_{d_{i}}\left(d_{t^{\prime }}\right)|d_{t^{\prime }}\in children\;of\;d_{t}\right\}, & d_{t}\neq d_{i}, \end{array},\right.$$ (1)

where γ is the coefficient regulating semantic contribution (Wang et al., 2010), and it was set to the optimal value of 0.5.

If two diseases have more overlaps in DAG, it implies greater similarity between them (Wang et al., 2010). Matrix $D_{S}\in R^{nd\times nd}$ represents the semantic similarity network of diseases, and its element $D_{S}\left(d_{i},d_{j}\right)$ represents the semantic similarity between diseases $d_{i}$ and $d_{j}$ .

$$D_{S}\left(d_{i},d_{j}\right)=\frac{\sum_{d_{m}\in \left(T_{d_{i}}\cap T_{d_{j}}\right)}\left(D_{d_{i}}\left(d_{m}\right)+D_{d_{j}}\left(d_{m}\right)\right)}{S\left(d_{i}\right)+S\left(d_{j}\right)},$$ (2)

where $T_{d_{i}}$ represents the DAG of disease $d_{i}$ and $S\left(d_{i}\right)$ represents the semantic value of disease $d_{i}$ .

$$S\left(d_{i}\right)=\sum_{d_{t}\in T_{d_{i}}}D_{d_{i}}\left(d_{t}\right).$$ (3)

LncRNA–lncRNA functional similarity network: Functionally similar lncRNAs are often associated with diseases in similar phenotypes (Wang et al., 2010). To calculate the functional similarity between two lncRNAs, the semantic similarity of diseases and its correlation to lncRNAs were utilized. Set $D=\left\{d_{1},d_{2},\cdots ,d_{t},\cdots ,d_{nd}\right\}$ represents the disease set, and $\max\left(d_{t},D\right)$ represents the maximum semantic similarity of any disease $d_{t}$ in set D :

$$\max\left(d_{t},D\right)=\max_{1\leq i\leq nd}\left(D_{S}\left(d_{t},d_{i}\right)\right).$$ (4)

Matrix $F_{S}\in R^{nl\times nl}$ represents the functional similarity network of lncRNAs, and matrix element $F_{S}\left(l_{i},l_{j}\right)$ represents the functional similarity between lncRNA $l_{i}$ and $l_{j}$ .

$$F_{S}\left(l_{i},l_{j}\right)=\frac{\sum_{1\leq i\leq m}\max\left(d_{i},D_{1}\right)+\sum_{1\leq j\leq n}\max\left(d_{j},D_{2}\right)}{m+n},$$ (5)

where set $D_{1}$ represents the set of diseases associated with lncRNA $l_{i}$ , set $D_{2}$ represents the set of diseases associated with lncRNA $l_{j}$ , and m and n represent the number of diseases in set $D_{1}$ and $D_{2}$ , respectively.

Gaussian interaction spectrum kernel similarity network: As an efficient and useful method in biological information classification, the Gaussian kernel function (Van Laarhoven et al., 2011) has been applied to the association network when some diseases do not have semantic similarity. Gaussian interaction spectrum kernel similarity of diseases (Gaussian similarity) calculated by the Gaussian kernel function could replace the semantic similarity of disease. If disease $d_{i}$ has a known experimentally verified association with any lncRNA, $I_{P}\left(d_{i}\right)=1$ ; if disease $d_{i}$ does not have any known association experimentally verified, $I_{P}\left(d_{i}\right)=0$ . Matrix $G_{D}\in R^{nd\times nd}$ represents the Gaussian similarity network of diseases, whose element $G_{D}\left(d_{i},d_{j}\right)$ represents the Gaussian similarity between disease $d_{i}$ and $d_{j}$ :

$$G_{D}\left(d_{i},d_{j}\right)=\exp\left(-\lambda_{d}{\left\|I_{P}\left(d_{i}\right)-I_{P}\left(d_{j}\right)\right\|}^{2}\right),$$ (6)

where $\lambda_{d}$ is the standardized core bandwidth, with detailed calculation as

$$\lambda_{d}=\frac{1}{\frac{1}{nd}\sum_{i=1}^{nd}{\left\|I_{P}\left(d_{i}\right)\right\|}^{2}}.$$ (7)

Similarly, matrix $G_{L}\in R^{nl\times nl}$ represents the Gaussian similarity network of lncRNAs, and matrix element $G_{L}\left(l_{i},l_{j}\right)$ represents the Gaussian similarity between lncRNA $l_{i}$ and $l_{j}$ .

$$G_{L}\left(l_{i},l_{j}\right)=\exp\left(-\lambda_{l}{\left\|I_{P}\left(l_{i}\right)-I_{P}\left(l_{j}\right)\right\|}^{2}\right).$$ (8)

$$\lambda_{l}=\frac{1}{\frac{1}{nl}\sum_{i=1}^{nl}{\left\|I_{P}\left(l_{i}\right)\right\|}^{2}}$$. (9)

Integrated similarity network: Since not all diseases involved could calculate the semantic similarity due to the inherent sparsity in the dataset, an integrated similarity network $D_{S}^{\left(I\right)}$ was constructed to improve the accuracy of disease semantic similarity. The matrix element $D_{S}^{\left(I\right)}\left(d_{i},d_{j}\right)$ was formed as

$$D_{S}^{\left(I\right)}\left(d_{i},d_{j}\right)=\left\{ \begin{array}{cc} D_{S}\left(d_{i},d_{j}\right)+G_{D}\left(d_{i},d_{j}\right), & D_{S}\left(d_{i},d_{j}\right)\neq 0,\\ G_{D}\left(d_{i},d_{j}\right), & D_{S}\left(d_{i},d_{j}\right)=0. \end{array}\right.$$ (10)

Similarly, matrix $F_{S}^{\left(I\right)}$ represents the integrated similarity network of lncRNAs, and the matrix element $F_{S}^{\left(I\right)}\left(l_{i},l_{j}\right)$ has the specific form as

$$F_{S}^{\left(I\right)}\left(l_{i},l_{j}\right)=\left\{ \begin{array}{cc} F_{S}\left(l_{i},l_{j}\right), & F_{S}\left(l_{i},l_{j}\right)\neq 0,\\ G_{L}\left(l_{i},l_{j}\right), & F_{S}\left(l_{i},l_{j}\right)=0. \end{array}\right.$$ (11)

Finally, a multi-source heterogeneous network as a diagonal matrix was constructed, preparing for the following calculation in the model:

$$X=\left[\begin{array}{cc} 0 & D_{S}^{\left(I\right)}\\ F_{S}^{\left(I\right)} & 0 \end{array}\right].$$ (12)

### Node feature enhancement

N-heads attention with multiple-operator aggregation: The original GAT utilizes attention scores to adaptively aggregate information from neighbor nodes during node updating and learns the representation of nodes on the graph by assigning different weights to its neighbor nodes. N-heads attention could stabilize the process of self-attention, with n time iterations (Fraidouni and Zaruba, 2019). However, n-heads attention only uses the “concatenation” operator to aggregate the features coming from each head. The aggregation effect needs to be improved further by adding more operators in each head, and a multiple-operator for n-heads attention was constructed to enhance node features.

Attention-based feature training: Any element in the feature vector matrix X was considered the node feature. In the kth iteration, attention score $e_{ij}^{k}$ of node i to neighbor node j in matrix X was calculated as

$$e_{ij}^{k}=f\left(h_{i}^{k}W,h_{j}^{k}W\right),$$ (13)

where f(⋅) denotes a single-layer neural network; $h_{i}^{k}$ denotes the feature vector of node i in the kth iteration; and $W\in R^{\left(nl+nd\right)\times 1}$ denotes the weighted matrix.

In order to make the attention score within the interval of [0,1], the softmax function was used for normalization

$$\alpha_{ij}^{k}=\frac{\exp\left(e_{ij}^{k}\right)}{\sum_{t\in N_{i}}\exp\left(e_{it}^{k}\right)},$$ (14)

where $N_{i}$ represents the set of all neighbor nodes of node i in matrix X . In the kth iteration, features of all nodes in set $N_{i}$ were calculated as

$$h_{N_{i}}^{k}=\sum_{t\in N_{i}}\alpha_{it}^{k}h_{t}^{k}.$$ (15)

**GNN-based feature aggregation:** In order to enhance node features further, based on a nonlinear graph neural network (GNN), a multiple-operator that aggregated the features coming from the attention-based feature training layer was designed:

$$\begin{array}{l} M^{k}=LeakyReLU\left(\left(h_{i}^{k}+h_{N_{i}}^{k}\right)W_{1}\right)+LeakyReLU\left(\left(h_{i}^{k}\|h_{N_{i}}^{k}\right)W_{1}\right)\\ +\left(LeakyReLU\left(\left(h_{i}^{k}+h_{N_{i}}^{k}\right)W_{1}\right)\times LeakyReLU\left(\left(h_{i}^{k}\|h_{N_{i}}^{k}\right)W_{1}\right))\right), \end{array}$$ (16)

where $M^{k}$ represents the feature vector after aggregating, LeakyReLU(⋅) is the activating function, “ + ” denotes the adding operation, “ ‖ ” denotes the concatenating operation, and $W_{1}\in R^{\left(nl+nd\right)\times k}$ is a weighted matrix. Finally, the feature vector $M^{k}$ *via* the n-heads attention mechanism formed the final feature matrix M :

$$M={\|}_{k=1}^{n}M^{k}=\left[\begin{array}{c} M^{d}\\ M^{l} \end{array}\right],$$ (17)

where $M^{d}\in R^{nd\times \left(nl+nd\right)}$ represents the feature matrix of diseases and $M^{l}\in R^{nl\times \left(nl+nd\right)}$ represents the feature matrix of lncRNAs.

### LncRNA–disease association reconstruction

Inductive matrix completion: Known LDA was represented as a low-rank matrix in original matrix completion which recovers missing elements only with less sampling data (Chen and Chen, 2017). However, a cold start phenomenon will occur, when the entire row or column of data is missing. IMC technology introduced could fix the cold start problem and improve prediction accuracy because the number of parameters that was learned in IMC only related to the number of features of lncRNAs (or diseases), not the number of lncRNAs (or diseases).

$${\hat{A}}_{ld}=M^{d}\gamma {\left(M^{l}\gamma \right)}^{T},$$ (18)

where ${\hat{A}}_{ld}$ represents the reconstruction of association matrix $A_{ld}$ and γ is the weight decay parameter.

Model optimization: Optimization of MM-LDA mainly focused on parameter training by minimizing the loss function. During parameter training, improper selection of learning rates will cause abnormal loss function. A large learning rate will lead to the non-convergence of the loss function. Otherwise, a small learning rate will make the model trap into local optimization. Therefore, the Adam optimizer (Kingma and Ba, 2014) that combined the advantages of an AdaGrad (adaptive gradient) optimizer (Lydia and Francis, 2019) and RMSprop (root mean square propagation) optimizer (Xu et al., 2021) was adopted in our model. Only requiring small memory space, the Adam optimizer with a simple and efficient implementation process could adjust the learning rate adaptively without being affected by gradient scaling, thus speeding up the model optimization speed. The optimization process by minimizing the loss function was formalized as

$$minLoss={{\left\|A_{ld}-{\hat{A}}_{ld}\right\|}_{F}^{2}+\lambda \left\|W_{2}\right\|}_{F}^{2},$$ (19)

where λ is the equilibrium factor with the value of 1 and $W_{2}\in R^{nl\times nd}$ denotes a weighted matrix.

## Results

### Experimental evaluation

Evaluation metrics: All known LDAs were randomly divided into five groups with which 5-fold cross-validation was carried out to evaluate the predictive performance of our model. Successively selecting one group in five (as negative samples) with a group of unknown lncRNA–disease pairs in the same size (as negative samples) made up the test samples. The remaining four groups in five and the remaining unknown lncRNA–disease pairs were used to train the model. A total of five model evaluation metrics were defined by setting different thresholds, including true positive rate (TPR), false positive rate (FPR), and recall rate. Model performance was measured by an area under the ROC curve (AUC) and an area under the PR curve (AUPR). In order to avoid the influence of grouping randomly, each experiment was repeated 10 times. Finally, an AUC value and AUPR value were calculated according to the average value of the results from the 10 repeated experiments.

Parameter selection: Parameters used in our model could impact the predictive performance in the process of model training. Therefore, this section discussed the selection process of these three parameters in detail.

**FIGURE 2:**
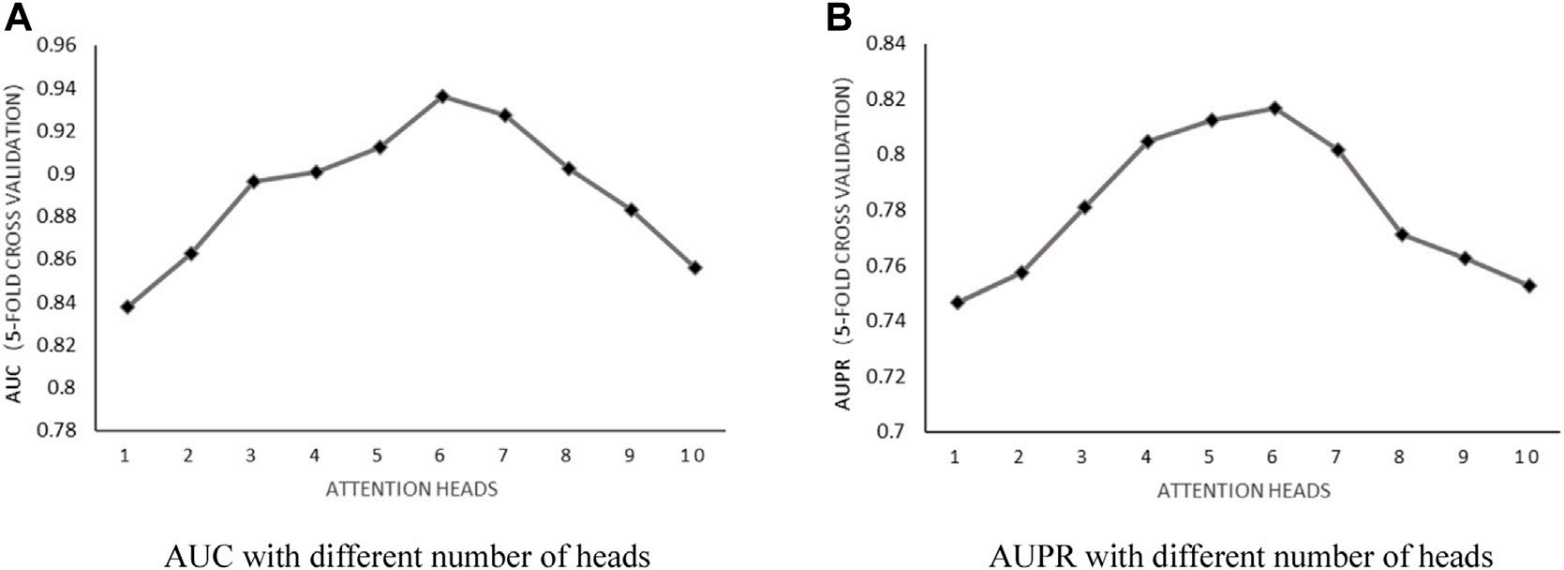
**(A)** AUC with different number of heads. **(B)** AUPR with different number of heads.

Number of attention heads: According to the literature (Fraidouni and Zaruba, 2019), the number of heads used in n-heads attention was discussed by setting the weight decay parameter γ as 5E-4 and the number of neurons as 8. After implementing 5-fold cross-validation, the results shown in Figure 2 proved that the number of heads impacted the predictive performance significantly. When the number of heads in n-heads attention was set to 6, the maximum AUC value and AUPR value could be obtained.

**FIGURE 3:**
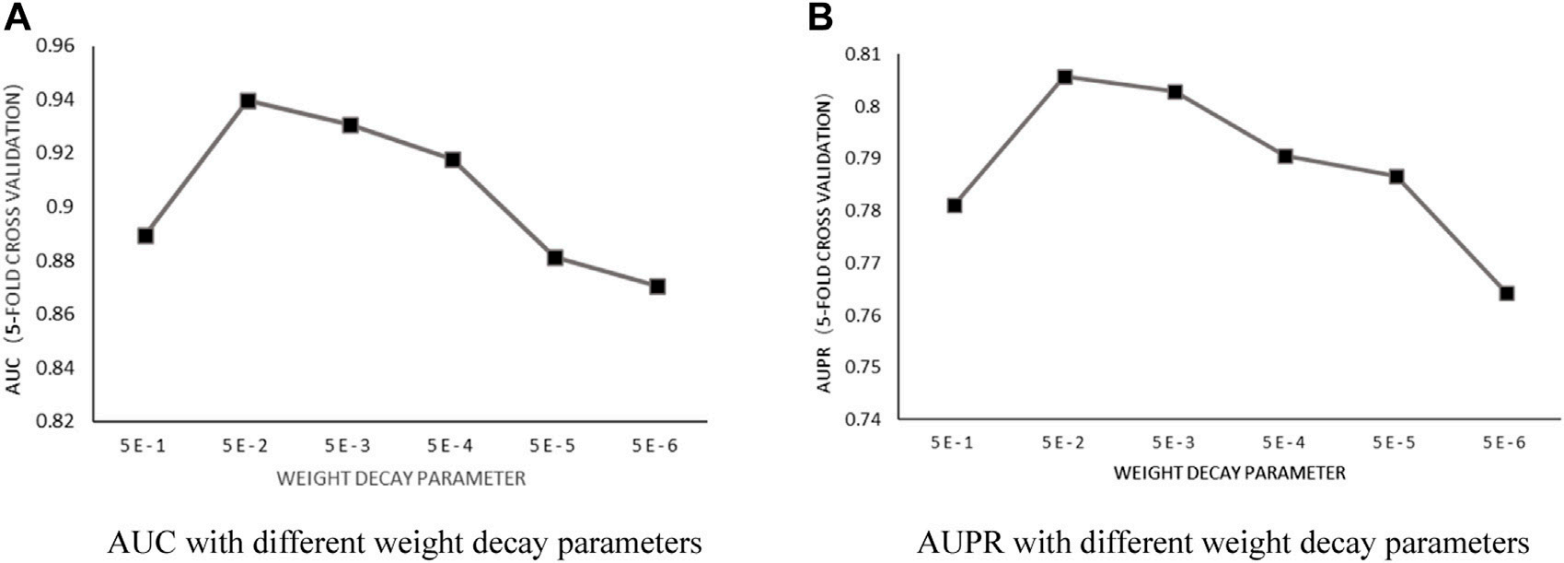
**(A)** AUC with different weight decay parameters. **(B)** AUPR with different weight decay parameters.

Weight decay parameter: According to the previous training, with the number of heads in a fixed value of 6 and the number of neurons in fixed value of 8, the influence of the weight decay parameter γ was discussed. The parameter value of γ was increased from 5E-6 to 5E-1, with a step size of E-1. After implementing 5-fold cross-validation, the results shown in Figure 3 proved that the model achieved the best predictive performance when γ was set to be 5E-2.

**FIGURE 4:**
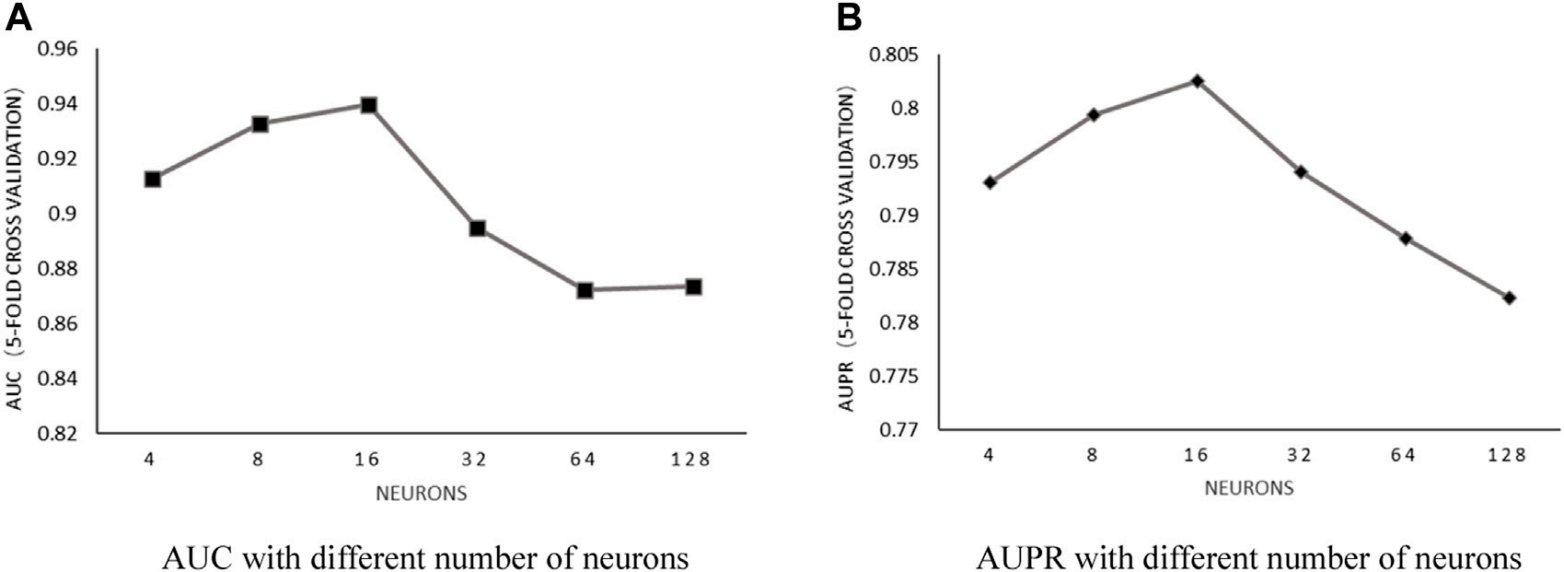
**(A)** AUC with different number of neurons. **(B)** AUPR with different number of neurons.

Number of neurons: With the number of heads in a fixed value of 6 and the weight decay parameter in fixed value of 5E-2, the influence of the number of neurons on predictive performance was discussed by choosing the value within the set of [4, 8, 16, 32, 64, and 128]. After implementing 5-fold cross-validation, the results shown in Figure 4 proved that AUC and AUPR obtained the best values when the number of neurons was set to 16.

Based on the previously mentioned discussion, by setting the number of heads in a fixed value of 6, the weight decay parameter γ in a fixed value of 5E-2, and the number of neurons in a fixed value of 16, our MM-LDA achieved the best AUC value of 0.9395 and AUPR value of 0.8057.

Ablation experiments: In order to evaluate the role of each kernel part in MM-LDA, such as multiple-operator aggregation in n-heads attention, IMC in lncRNA–disease association reconstruction, three ablation experiments that were used to compare with our MM-LDA were set up:

• GAT-NG: A prediction model was constructed without kernel similarity of the Gaussian interaction spectrum as the kernel part.
• GAT-GIMC: A prediction model was constructed only based on a standard multiple-heads graph attention network.
• GAT-GMC: A prediction model was constructed only based on standard matrix completion.

**FIGURE 5:**
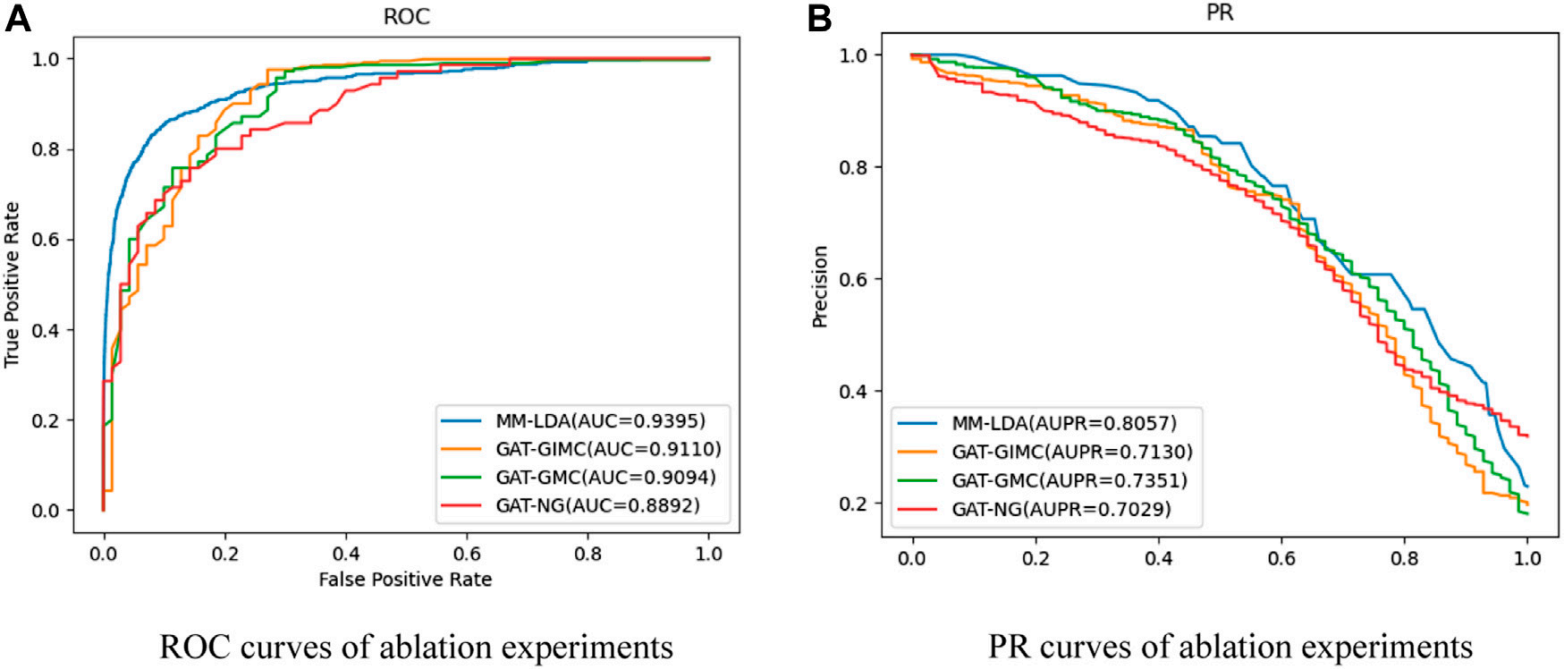
**(A)** ROC curves of ablation experiments. **(B)** PR curves of ablation experiments.

For each ablation experiment, 5-fold cross-validation was repeated 10 times, and the average values of the results are shown in Figure 5.

From the results shown, MM-LDA obtained 5.65%, 3.3%, and 3.1% higher AUC values than GAT-NG, GAT-GMC, and GAT-GIMC, respectively. Furthermore, MM-LDA obtained 14.62%, 9.6%, and 13% higher AUPR values than GAT-NG, GAT-GMC, and GAT-GIMC, respectively. Therefore, it proved that the three kernel parts (integrated Gaussian interaction spectrum kernel similarity, multiple-operator aggregation in n-heads attention, and IMC) of MM-LDA could significantly improve the predictive performance.

**FIGURE 6:**
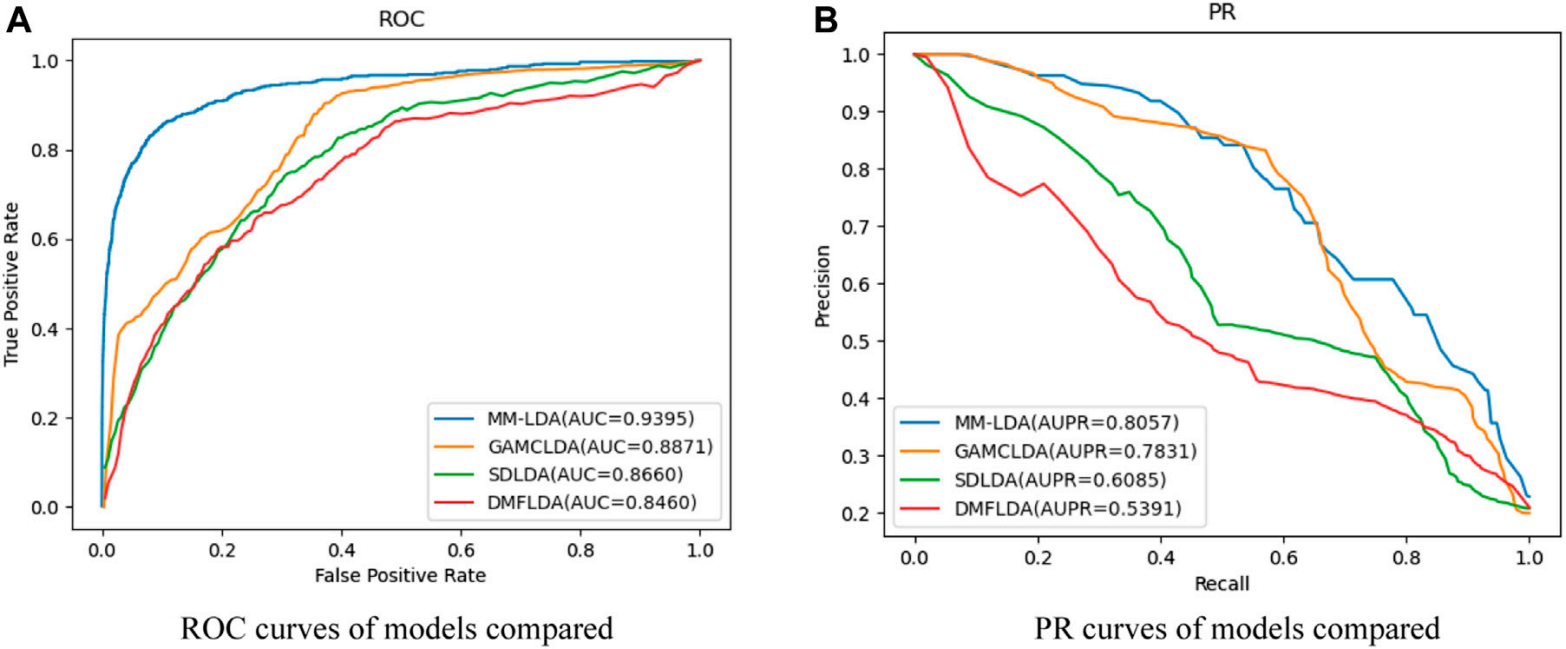
**(A)** ROC curves of models compared. **(B)** PR curves of models compared.

Comparison with other models: SDLDA (Zeng et al., 2020b), DMFLDA (Zeng et al., 2020a), and GAMCLDA (Lu et al., 2019), the three computational models based on machine learning and matrix factorization in recent 3 years, were compared with our MM-LDA on the same dataset ( $A_{ld}\in R^{nl\times nd}$ ). After 5-fold cross-validation was carried out, the detailed results are shown in Figure 6 and Table 1 to further prove the remarkable performance of MM-LDA.

**TABLE 1:** AUC value (AUPR value) and running time of models compared.

Model	AUC	AUPR	Time (hour)
MM-LDA	0.9395	0.8057	1.24
GAMCLDA	0.8871	0.7831	1.32
SDLDA	0.8660	0.6085	1.15
DMFLDA	0.8460	0.5391	1.18

From the results shown, we could easily find that MM-LDA obtained the best AUC value that is 5.9%, 6.05%, and 11.05% higher than that of GAMCLDA, SDLDA, and DMFLDA, respectively. In addition, MM-LDA also obtained the best AUPR value that is 2.9%, 32.4%, and 49.5% higher than that of GAMCLDA, SDLDA, and DMFLDA, respectively. Though the running time of MM-LDA is 7.82% and 5.08% longer than that of SDLDA and DMFLDA, MM-LDA achieved the highest cost-effective prediction performance comprehensively.

## Case study

In order to further verify the independent prediction performance of MM-LDA, gastric cancer was selected as the target for the case study. All known associations relating to gastric cancer composed the training set, and unknown associations composed the testing set. Then, gastric cancer-related lncRNAs identified by MM-LDA were sorted by scores. The top 10 lncRNAs with the highest scores were selected to validate the predictive performance of MM-LDA, with the evidence coming from relevant literature and database, as shown in Table 2.

**TABLE 2:** Top 10 gastric cancer-related lncRNAs.

Rank	LncRNA	Evidence
1	UCA1	LncRNA disease
2	TCL6	Literature [6]
3	PCA3	Literature [6]
4	HOTAIR	LncRNA disease
5	H19	LncRNA disease
6	MALAT1	Unconfirmed
7	BCAR4	LncRNA disease
8	HCP5	LncRNA disease
9	CDKN2B-AS1	LncRNA disease
10	HTTAS	Unconfirmed

In Table 2, all but two out of 10 lncRNAs predicted by MM-LDA have found evidence from relevant literature and database. Even though, there is no direct evidence showing that HOTAIR and HTTAS relate to gastric cancer so far, some studies found that HOTAIR has stable expression in peripheral blood and can be used as a non-invasive diagnostic marker for gastric cancer (Dong et al., 2019). There is also no published literature which finds the association between HTTAS and gastric cancer. We firmly believe that there will be some researchers to find the experimental evidence for this association inferred by MM-LDA.

## Discussion

In this study, a new lncRNA–disease association prediction model, namely, MM-LDA, combining the graph attention network and inductive matrix completion technology was established. MM-LDA designed a multiple-operator aggregation in n-heads attention to enhance the features of nodes. The enhanced features were input into the whole process of induction matrix completion, and the original association matrix was reconstructed by completing the missing elements of the matrix. The results from 5-fold cross-validation showed that MM-LDA obtained the best AUC value and AUPR value compared with the other three state-of-the-art computational models. Comparing with GAMCLDA, 6.45% of training time was saved. In general, MM-LDA deserves to be recommended as the highest cost-effective prediction model. However, there are still some aspects that need to be further improved and studied. First, more biological information relating to lncRNAs and diseases should be effectively integrated. Second, MM-LDA did not predict the associations relating to new lncRNAs and isolated diseases because we could not capture the features of new lncRNAs and isolated diseases without known associations. Third, we should continue to optimize the aggregators by considering the research progress of association prediction in other fields.

## Data Availability

The original contributions presented in the study are included in the article/Supplementary Material; further inquiries can be directed to the corresponding author.

## Abbreviations

ROC, receiver operating characteristic; AUC, area under the ROC curve; FPR, false positive rate; TPR, GAT, IMC, and LDA true positive rate, graph attention network, inductive matrix completion, and LncRNA–disease associations, respectively.
